# Inflammatory activation of surface molecule shedding by upregulation of the pseudoprotease iRhom2 in colon epithelial cells

**DOI:** 10.1038/s41598-021-03522-2

**Published:** 2021-12-20

**Authors:** Anja Adelina Giese, Aaron Babendreyer, Peter Krappen, Annika Gross, Pavel Strnad, Stefan Düsterhöft, Andreas Ludwig

**Affiliations:** 1 Institute of Molecular Pharmacology, Medical Faculty, RWTH Aachen University, Pauwelsstr. 30, 52074 Aachen, Germany; 2 Division of Gastroenterology and Hepatology, Department of Medicine III, University Hospital RWTH Aachen, Aachen, Germany

**Keywords:** Proteases, Mechanisms of disease, Chronic inflammation, Interferons, Tumour-necrosis factors

## Abstract

The metalloproteinase ADAM17 contributes to inflammatory and proliferative responses by shedding of cell-surface molecules. By this ADAM17 is implicated in inflammation, regeneration, and permeability regulation of epithelial cells in the colon. ADAM17 maturation and surface expression requires the adapter proteins iRhom1 or iRhom2. Here we report that expression of iRhom2 but not iRhom1 is upregulated in intestinal tissue of mice with acute colitis. Our analysis of public databases indicates elevated iRhom2 expression in mucosal tissue and epithelial cells from patients with inflammatory bowel disease (IBD). Consistently, expression of iRhom2 but not iRhom1 is upregulated in colon or intestinal epithelial cell lines after co-stimulation with tumor necrosis factor (TNF) and interferon gamma (IFNgamma). This upregulation can be reduced by inhibition of Janus kinases or transcription factors NF-kappaB or AP-1. Upregulation of iRhom2 can be mimicked by iRhom2 overexpression and is associated with enhanced maturation and surface expression of ADAM17 which then results in increased cleavage of transforming growth factor (TGF) alpha and junctional adhesion molecule (JAM)-A. Finally, the induction of these responses is suppressed by inhibition of iRhom2 transcription. Thus, inflammatory induction of iRhom2 may contribute to upregulated ADAM17-dependent mediator and adhesion molecule release in IBD. The development of iRhom2-dependent inhibitors may allow selective targeting of inflammatory ADAM17 activities.

## Introduction

Inflammation and subsequent tissue repair are characterized by the increased presence of soluble mediators. These soluble mediators comprise cytokines, cytokine receptors, growth factors and adhesion molecules^1–5^. Several of the soluble mediators originate from limited proteolysis at a membrane-proximal, extracellular site within the membrane-anchored precursor of these mediators. This process is termed shedding and, in many cases, it involves the activity of the a disintegrin and metalloproteinase (ADAM) family members ADAM10 and ADAM17^6–10^. Key substrates for ADAM17 include the tumor necrosis factor (TNF) α^11–13^, the transforming growth factor (TGF) α^14^ and the junctional adhesion molecule (JAM)-A^8^. These mediators are critically implicated in inflammatory and repair functions in vivo*.* Especially TNFα is well known to drive inflammatory bowel disease (IBD)^15^. TGFα contributes to epithelial tissue regeneration^16^ and JAM-A regulates permeability and inflammation in the intestine^17^. The importance of ADAM17-mediated shedding events in the intestine is indicated by analysis of transgenic mice in models of intestinal inflammation. On the one hand, hypomorphic mice with a reduced ADAM17 activity suffer from defective regeneration of epithelial cells via growth factors such as TGFα and breakdown of the intestinal barrier^18^. On the other hand, a complete, cell specific loss of ADAM17 in intestinal epithelial cells attenuates TNFα driven mucosal atrophy in a mouse model of parenteral nutrition^19^. Thus, ADAM17 can promote both inflammatory and regenerative processes.

The importance of ADAM17 in various pathologies as well as repair functions necessitates that the protease is tightly regulated. ADAM17 is expressed constitutively in almost all cell types and its expression can be upregulated to some degree^20,21^. Notably, ADAM17 is not only expressed at the cell surface but present in considerable amounts inside the cells. Moreover, overexpression is not sufficient for enhanced ADAM17-mediated shedding, indicating that further posttranslational control mechanisms need to be in place before ADAM17 activity can be increased^22^. First, ADAM17 is synthesized into the endoplasmic reticulum (ER) as an immature form in which the metalloproteinase activity is blocked by a prodomain^23,24^. In the ER immature ADAM17 interacts with iRhom1 or 2 which are inactive members of the rhomboid family ^25–28^. This interaction is required for ADAM17 trafficking to the Golgi apparatus where the prodomain of the protease is proteolytically removed, before the mature protease is then transported to the cell curface^23^. Additionally, iRhoms are necessary for the sustained expression and activity of ADAM17 on the cell surface^29–31^. Here mature ADAM17 can be activated by further mechanisms^32,33^, including conformational changes orienting the protease activity close to the membrane to cleave its substrates^34^.

So far, iRhom1 and 2 appear to be specialized molecules for controlling ADAM17 maturation and surface expression. Notably, iRhom2 is predominantly found in immune cells while iRhom1 is expressed in most parenchymal tissue cells^35^. Consistent with this expression profile it was found that iRhom2 is critically involved in immune functions in models of acute and chronic inflammation but is less important for physiologic functions of the tissue^33,36,37,37–39^. By contrast iRhom1 has been linked to critical homeostatic functions^26,38–40^.

We here address the question, whether iRhom2 is only relevant in leukocytes or also of importance in parenchymal tissue cells. To this end, we investigate expression of both iRhoms in murine tissue from a model of colon inflammation as well as in human colon or intestinal epithelial cell lines. We describe cytokine driven transcriptional pathways that lead to upregulation of iRhom2 but not iRhom1 in vitro, and we demonstrate the consequences of this upregulation for ADAM17 maturation, surface expression and shedding of epithelial surface molecules.

## Materials and methods

### Antibodies and reagents

Unconjugated mouse monoclonal antibody (mab) against ADAM17 ectodomain (clone 111,633 MAB-9301), mouse mab isotype control (Clone 11,711) and mouse mab against iRhom2 (Clone996308) were from R&D (Minneapolis, USA). Allophycocyanin (APC)-coupled IgG_1_ was from Jackson ImmunoResearch (Hamburg, Germany). Rabbit mab against ADAM17 (C-terminus) was from Millipore (Darmstadt, Germany). Mouse mab against GAPDH (GA1R) was from Thermo Scientific (Waltham, MA USA). Peroxidase (POD)-conjugated secondary antibodies were from Jackson ImmunoResearch (Hamburg, Germany). Tumor necrosis factor α (TNFα) and Interferon γ (IFNγ) were from PeproTech (Rocky Hill, USA). Nuclear factor kappa-light-chain-enhancer of activated B cells (NF-κB) inhibitor Bay11-7082 and Activator protein 1 (AP-1) inhibitor SR 11,302 were from Santa Cruz Biotechnology (Dallas, USA) and JAK 1 and 2 inhibitor Baricitinib was from Selleck Chemicals (Houston, USA).

### Animal tissues

The colitis experiments with 10 weeks old C56 Bl 6 mice had been performed as previously described^41^ and reversely transcribed cDNA was provided for mRNA expression analysis. In brief, mice were infected with 1 × 10^9^ *C. rodentium* and sacrificed 14 d after infection. In another series of experiments, mice treated for a short term with dextran sodium sulfate (DSS) were given drinking water with 1.6% DSS for 4 d and sacrificed on day 4. Long term treated mice received drinking water containing 1.6% DSS for 5 days, followed by 2 days of tap water and then sacrificed on day 7. The animal experiments were approved by the state of North Rhine-Westphalia in Germany and the University of Aachen animal care committee and were conducted in compliance with the German Law for Welfare of Laboratory Animals and with the the ARRIVE guidelines.

### Bioinformatic analyses

Public transcriptomic mRNA expression data of human samples generated by Affymetrix Human Genome U133 Plus 2.0 Array were obtained from a variety of public repositories and analyzed with the tool GENEVESTIGATORv8 suite (Nebion AG, Zürich, Switzerland)^42^.

The promotor region of iRhom2 was narrowed down to -1500 bp TSS + 500 bp by identifying CpG islands upstream of the transcription start site using the UCSC Genome Browser (UCSC, Santa Cruz, USA)^43^. The analysis of potential binding sites within this region was performed with CiiiDER(Hudson Institute of Medical Research, Melbourne, AUS)^44^.

### Cell culture, transduction, cytokine stimulation, inhibitor treatment and transient transfection

The human epithelial colorectal adenocarcinoma cell lineHT-29 (ATCC HTB-38), the human intestinal subclone TC7 of Caco-2 cells (ATCC HTB-37) and the lung adenocarcinoma cell line A549 (ATCC CCL185) were cultured in DMEM medium with 10% FCS and 1% Penicillin/Streptomycin.

HT-29 cells were transduced to express iRhom2. For overexpression, cDNA encoding iRhoms (TransOMIC Technologies Inc., Huntsville, USA), was amplified by PCR and inserted into the viral expression vector pLVX-IRES-Puro. Insertion was performed with the restriction enzymes EcoRI and NotI for iRhom2 cDNA, and EcoRI and XhoI for iRhom1 cDNA. Recombinant lentiviruses were produced as described before^45^. For transduction 1 × 10^5^ cells were seeded into six-wells, and concentrated lentivirus preparation (5 µl) was added directly after seeding. To enhance the transduction efficiency, polybrene (8 µg/ml, Sigma) was added. After 24 h, cells were washed and further cultivated in complete cell culture medium for another 24 h. Transduced cells were selected by cultivation in medium containing Puromycin (100 ng/ml) and kept in selection-medium until treatment.

For stimulation experiments, cells were grown in six-wells at a density of 2.5 × 10^5^ for 24 h. Then, TNFα or IFNγ or a combination of both (each at concentration of 10 ng/ml) were added and the cells were incubated for the indicated time periods before they were cooled on ice for further analysis.

For inhibitor treatment, cells received either Baricitinib (1 µM), Bay11-7082 (3 µM), or SR 11,302 (3 µM) 1 h prior to stimulation with TNFα and/or IFNγ.

Transient transfection with a plasmid pcDNA 3.1 with TGFα N-terminally fused to an alkaline phosphatase was performed with Lipofectamine 3000 (Thermo; L3000015) following the manufacturer’s protocol (Lipofectamine™ Reagent protocol).

### Flow cytometric analysis

Cells were stained with anti-ADAM17-ectodomain antibody (1 µg/ml) in PBS supplemented with 0.2% BSA for 1 h on ice. Isotype control for IgG1, was used in parallel. Detection of bound antibodies was performed using APC-conjugated anti-mouse antibody (1:500). The fluorescence signal was then analyzed by flow cytometry (LSRFortessa, BD Biosciences, Heidelberg, Germany) and the median fluorescence intensity (MFI) was determined with FlowJo 8.7.3 software (Tree Star, Inc., Ashland, USA) as quantitative parameter. The unspecific MFI determined for the isotype control was subtracted from the total MFI to obtain the specific MFI for ADAM17 surface expression.

### Quantitative PCR analysis

The mRNA expression levels of ADAM17, ADAM10, iRhom1 and iRhom2 were measured by quantitative real-time PCR and normalized in cell culture samples to the mRNA expression level of glyceraldehyde-3-phosphate dehydrogenase (GAPDH) and in murine tissue samples to the mRNA expression level of ribosomal protein L7(mL7). The general procedure has be described previously^46^. RNA of murine tissue samples was extracted using RNeasy Kit (Qiagen, Hilden, Germany). RNA extraction of cell culture samples was performed using Extractme Total RNA Kit (blirt, Gdansk, Poland). Both types of RNA samples were quantified (NanoDrop, Peqlab, Erlangen, Germany). RNA (250 ng) was reverse transcribed using RevertAid First Strand cDNA Synthesis Kit (Fermentas, St Leon-Rot, Germany) according to manufacturers’ protocols. PCR reactions were then performed in duplicates of 10 µl volume containing 1 µl of cDNA template, 5 µl 2 × SYBR Premix Ex Taq II (Takara, Fitchburg, WI USA), 3 µl H_2_O and 0.5 µM forward and reverse primer. Following primers were *used for murine tissue samples: mAdam17 forward aaaccagaacagacccaacg; mADAM17 reverse gtacgtcgatgcagagcaaa; mAdam10 forward agcaacatctggggacaaac; mAdam10 reverse tggccagattcaacaaaaca; miRhom2 forward agagcgtgaagtacatcc; miRhom2 reverse taaagtctccgagcagtcc; miRhom1 forward ttcttcacttactggctcac; miRhom1 reverse ttccgaagtaccgagtcc; mL7 forward tggaaccatggaggctgt; mL7 reverse cacagcgggaacctttttc. Following primers were used for human cell culture samples: ADAM17 forward, gaagtgccaggaggcgatta; ADAM17 reverse, cgggcactcactgctattacc; ADAM10 forward ggattgtggctcattggtgggca ADAM10 reverse actctctcggggccgctgac, iRhom2 forward cgattgacctgatccacc, iRhom2 reverse caaagtctccgagcagtcc, iRhom1 forward gacagcccacatctcttcac, iRhom1 reverse tccttgctcactccaaaccca. GAPDH forward, ccagccccagcgtcaaaggtg; GAPDH reverse, agggccgatcatggagtctt*. All PCR reactions were run on a CFX Connect Real-Time PCR Detection System (Bio-Rad) with the following protocol: 40 cycles of 10 s denaturation at 95 °C, followed by 10 s annealing at the indicated temperature (mAdam17 (57 °C); mAdam10 (57 °C); miRhom2 (60 °C); miRhom1 (60 °C); mL7 (55 °C); ADAM17 (55 °C); ADAM10 (61 °C); iRhom2 (58 °C); iRhom1 (56 °C); GAPDH (66 °C); L7 (60 °C)) and 15 s amplification at 72 °C. PCR efficiency was determined from the uncorrected RFU values using LinRegPCR version 2020.0 (11). Relative quantification was performed with the CFX Maestro Software 1.1 (Bio-Rad).

### Western blotting

The general procedure of Western Blot detection of ADAM17 and iRhom2 has been described previously^46^. Samples from cell lysates were incubated in SDS sample buffer (250 mM Tris HCl (pH 6.8), 50% (w/v) glycerol, 10% (w/v) SDS, 0.1% bromophenol blue and 5% β-mercaptoethanol) for 30—45 min at room temperature and subjected to SDS–polyacrylamide gel electrophoresis using 10–12.5% Tris–glycine gels. Proteins were transferred onto polyvinylidene difluoride membranes (Hybond-P, Amersham). Membranes were blocked with 5% (w/v) non-fat dry milk in tris buffered saline with 0.05% Tween for 1 h and probed with primary antibodies against ADAM17 (1 µg/ml), iRhom2 (1 µg/ml) or GAPDH (1 µg/ml) for 1 h or overnight at 4 °C followed by incubation with HRP-coupled secondary antibodies (diluted 1:20.000). Equal loading and transfer of proteins to the membrane was verified by detection of GAPDH using a specific monoclonal antibody. After addition of chemiluminescence substrate (ECL advanced, Amersham), signals were recorded using a luminescent image analyzer LAS3000 and Multi Gauge 3.0 software (Fujifilm, Tokyo, Japan) and quantified by using the open-source Image Studio Lite software (LI-COR Biosciences). ADAM17 was detected as two protein bands representing the mature form of ADAM17 (mADAM17) and the proform of ADAM17 (pADAM17) of ~ 100 and 130 kDa, respectively. iRhom2 was detected as one band of ~ 90 kDa and GAPDH as one band of ~ 37 kDa. Densitometric analysis was performed with Image Studio Lite (Li-Cor, Lincoln, NB, USA). ADAM17 total protein (tADAM17) was determined as signal density of pADAM17 and mADAM17 together. Protein levels were then expressed as signal density ratio of either tADAM17 or iRhom2 and GAPDH or pADAM17 and mADAM17.

### JAM-A and IL-8 release

Unstimulated or cytokine-stimulated cells were incubated for 24 h. Conditioned media were harvested and cleared by centrifugation (10 min, 4 °C; 16 000 g). Released soluble, JAM-A (JAM-A ELISA kit, SinoBiological, Beijing, China) or IL-8 (Human IL-8/CXCL8 DuoSet ELISA, R & D Systems, Minneapolis, USA) were quantified per ELISA essentially as recommended by the manufacturers. The chromogenic reaction was mediated by a standard procedure using 0.1 U/ml streptavidin-conjugated horseradish peroxidase (Roche, Basel, Switzerland) and the BM Blue POD substrate (Roche).

### Shedding activity

Shedding activity of ADAM17 was measured by an alkaline phosphatase (AP)–based assay as described before^47^. In brief, 1.5 × 10^5^ cells were seeded in 12-wells and transiently transfected with the ADAM17 substrate TGFα fused to an alkaline phosphatase (AP). After 4 h cells were washed, and fresh complete cell culture medium was added. After another 20 h cells were treated with cytokines or inhibitors, or both as mentioned above. The shedding activity was assessed by measuring the AP activity in the supernatant and in cell lysates (lysis buffer: 50 mM Tris; 137 mM NaCl; 2 mM EDTA; 10 mM 1,10-Phenanthroline; pH7.5). By adding p-Nitrophenyl phosphate (PNPP) solution (Thermo; 37,620) the AP activity could be continuously measured at 405 nm with the FLUOstar Optima (BMG LABTECH, Ortenberg, Germany)). To assess the AP activity the slope (change of absorption at 405 nm/ms) was calculated. The amount of ADAM17 activity was calculated as PNPP substrate turnover (AP activity) in the supernatant in relation to the total turnover in supernatant plus cell lysate.

### Statistics

Quantitative data are shown as mean plus SD calculated from at least three independent experiments, n numbers are specified in the figure legends. Statistics were performed using the generalized mixed model analysis (PROC GLIMMIX, SAS 9.4, SAS Institute Inc., Cary, North Carolina, USA) and assumed to be from either normal, lognormal or beta distribution with the day of experiment conduction as random to assess differences in the size of treatment effects across the results. Residual analysis and the Shapiro–Wilk test were used as diagnostics. In the case of heteroscedasticity (according to the covtest statement) the degrees of freedom were adjusted by the Kenward-Roger approximation. All p-values were adjusted for multiple comparisons by the false discovery rate (FDR).

### Ethics approval and consent to participate

We herewith confirm that all methods were carried out in accordance with relevant guidelines and regulations.

## Results

### iRhom2 but not iRhom1 is transcriptionally upregulated in a colitis model

Tissue cells are known to express iRhom1 under basal conditions while immune cells preferentially express iRhom2^26,35^. To get an idea of the expression level in the healthy colon, we performed a bioinformatic analysis of the reported expression data for different colon epithelial tissues using GENEVESTIGATOR®^42^. This analysis revealed that iRhom1 mRNA is highly expressed in these tissues under basal conditions, whereas iRhom2 mRNA is expressed at considerably lower levels (suppl. Figure 1). Of note, ADAM17 is also highly expressed in these tissues.

**Figure 1:**
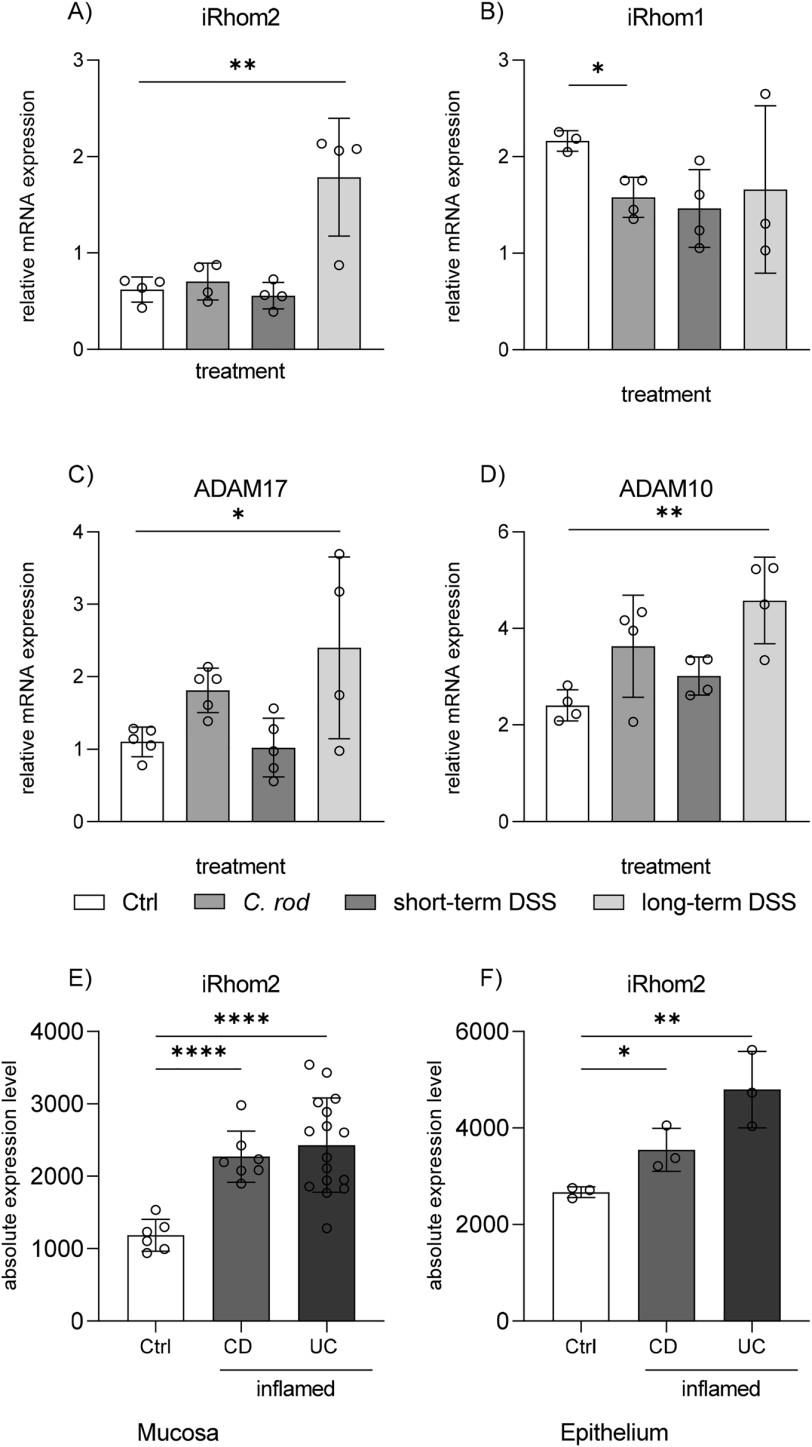
Long-term treatment with DSS leads to induced mRNA expression of iRhom2 and ADAM17 in murine colonic tissue. (**A**–**D**) Colon tissue was used from different murine models of colon inflammation. Mice had been either infected with *C. rodentium* or treated with DSS in drinking water for a short-term or for long-term, respectively. Colon tissues were analyzed for relative mRNA expression of iRhom2 (**A**), iRhom1 (**B**), ADAM17 (**C**) and ADAM10 (**D**) with mL7 as reference gene by qPCR. (**E**–**F**): Analysis of public transcriptome data from human colon tissue samples revealed that iRhom2 is upregulated in mucosal tissue (**E**) and colon epithelial cells (**F**) from patients with Crohn's disease (CD) or ulcerative colitis (UC). Data are shown as mean + SD of at least three independent experiments. Statistical differences in comparison to the control (Ctrl) are indicated by asterisks (* = p ≤ 0.05; ** = p ≤ 0.01; *** = p ≤ 0.001, **** = p ≤ 0.001).

To further investigate the possible regulation of both iRhoms under inflammatory conditions we used colonic tissues previously obtained from different murine colitis models to study the mRNA expression of iRhom1 and 2. In these models colitis had been induced by either bacterial infection with *C. rodentium*, 4 days of DSS supply with drinking water (short-term model) or 5 days of DSS supply plus 2 days without DSS (long-term model)^41^.We found that mRNA expression of iRhom2 but not iRhom1 is increased threefold in the long-term DSS model (Fig. 1A,B). Also, ADAM17 and ADAM10 mRNA expression was upregulated almost twofold under these conditions (Fig. 1C,D). Notably neither the bacterial infection nor the short-term exposure with DSS were sufficient to increase iRhom2 mRNA expression. Further analysis of transcriptome data from human colon tissue samples revealed that iRhom2 is upregulated in mucosal tissue and colon epithelial cells from patients with Crohn's disease (CD) or ulcerative colitis (UC) (Fig. 1E,F).

### Cytokines upregulate iRhom2 but not iRhom1 in a gut epithelial cell line

**Figure 2:**
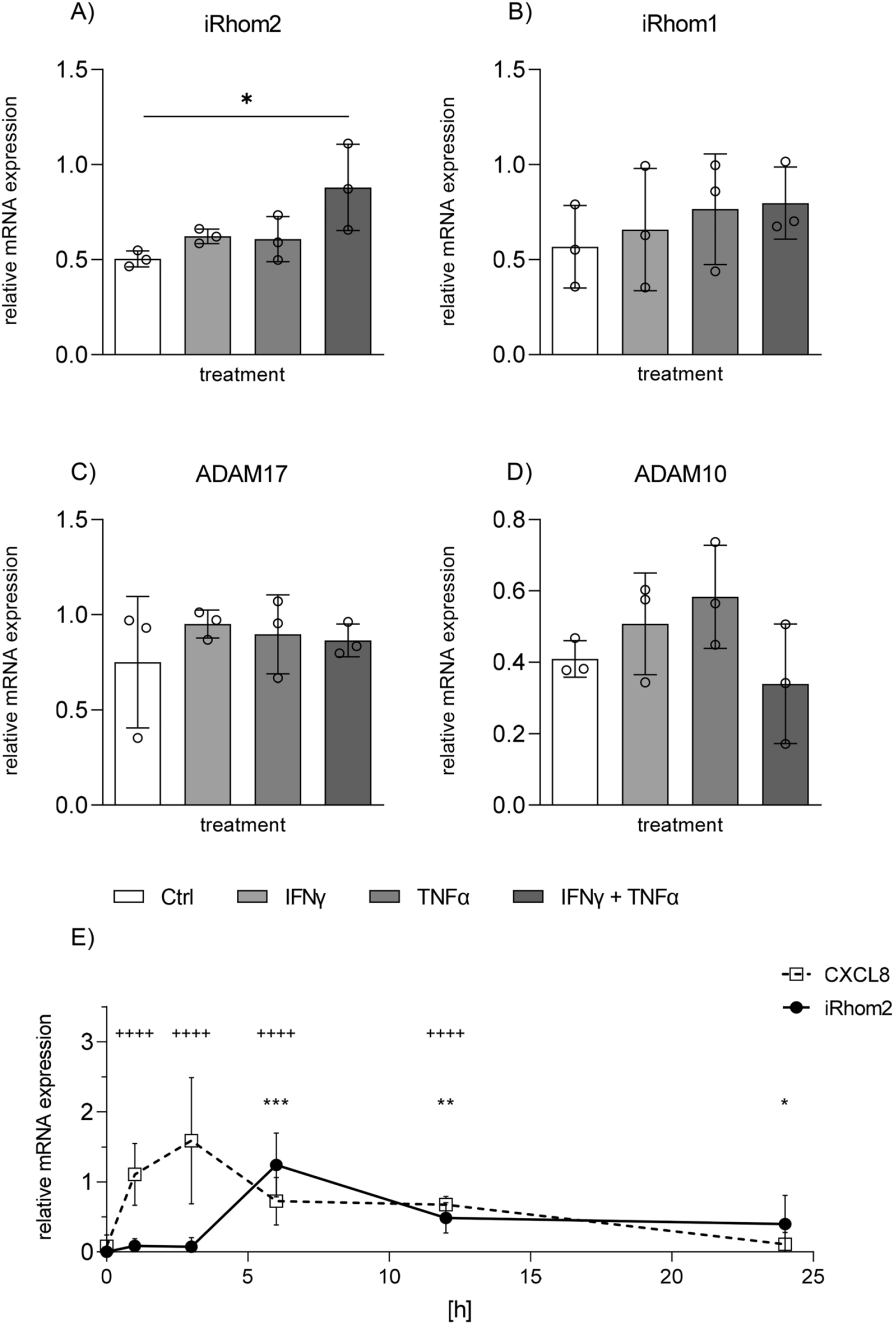
Stimulation with IFNγ and TNFα leads to an induced expression of iRhom2 in HT-29 cells. HT-29 cells were left unstimulated (Ctrl) or stimulated with IFNγ, TNFα or both (each cytokine 10 ng/ml) for 24 h. Afterwards, cells were harvested and analyzed by qPCR for relative mRNA expression of iRhom2 (**A**), iRhom1 (**B**), ADAM17 (**C**) and ADAM10 (**D**) with GAPDH as reference gene. HT-29 cells were stimulated with a combination of IFNγ and TNFα (each cytokine 10 ng/ml) for the indicated time periods. Subsequently, mRNA expression of iRhom2 and IL-8 was analyzed by qPCR with GAPDH as reference gene. The mRNA expression shown indicates the expression of CXCL8 or iRhom2 minus the basal expression at each indicated time point (E). Data are shown as mean + SD of at least three independent experiments. Statistical differences in comparison to the control (Ctrl) are indicated by asterisks for iRhom and crosses for CXCL8 (* = p ≤ 0.05; ** = p ≤ 0.01; *** = p ≤ 0.001).

To study further whether iRhom2 can be upregulated in vitro we chose the human colon epithelial cell line HT-29. As stimuli we used the cytokines IFNγ and TNFα which are well known to act as critical proinflammatory mediators during the course of colitis^48^. Cells were either treated with IFNγ or TNFα alone or in combination. We observed a detectable basal expression of iRhom1 and iRhom2 in these cells (Fig. 2A,B). The iRhom2 mRNA expression was not increased by IFNγ or TNFα alone but was increased by a combination of both cytokines. (Fig. 2A). In contrast, iRhom1 was not upregulated by the cytokines either alone or in combination (Fig. 2B). The expression of ADAM17 mRNA and ADAM10 mRNA were also not affected (Fig. 2C,D). Thus, IFNγ and TNFα seem to cooperate to specifically enhance the iRhom2 expression in gut epithelial cells.

We next studied the time kinetic of iRhom2 upregulation in response to co-stimulation with IFNγ and TNFα. Interestingly, the induction turned out to be transient with a sharp peek at the 6 h time point and at 12 h the expression again declined (Fig. 2E). By contrast induction of CXCL8 expression was more rapid with a peak at 1 h and declining thereafter . Neither the gene expression of iRhom1, ADAM17 nor ADAM10 was induced by co-stimulation over the investigated time period (suppl. Figure 2.).

### Upregulation of iRhom2 leads to increased maturation and surface expression of ADAM17

**Figure 3:**
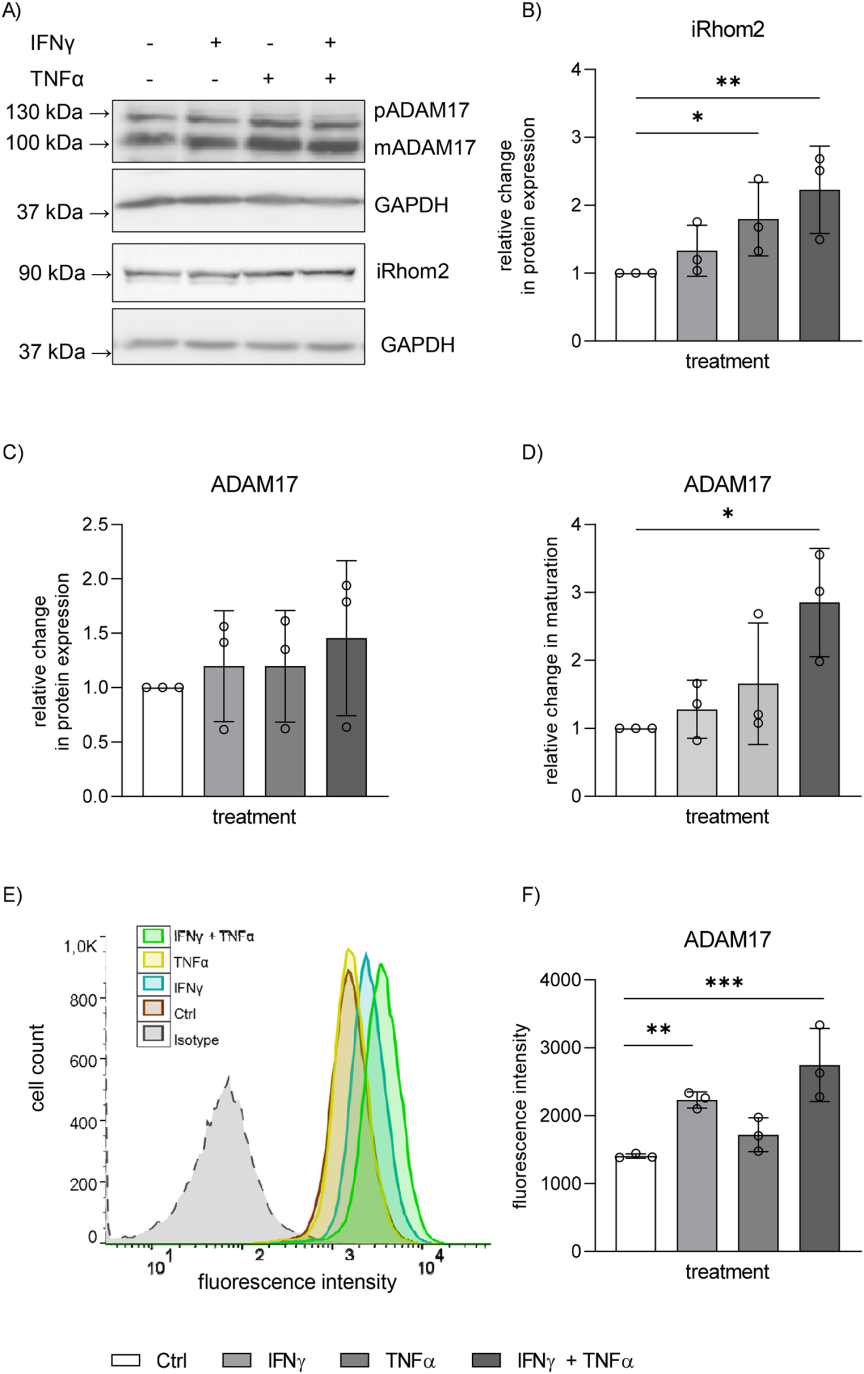
Co-stimulation with IFNγ and TNFα leads to increased iRhom2 protein expression, ADAM17 maturation and surface expression. HT-29 cells were left unstimulated (Ctrl) or stimulated with IFNγ, TNFα or both (each cytokine 10 ng/ml) for 24 h. (**A**–**D**): Subsequently, protein levels of iRhom2 and ADAM17 were analyzed by western blot with GAPDH as loading control. Exemplary western blots of iRhom2, ADAM17 and GAPDH are shown (**A**). Full western blot images are provided in suppl. (**A**) Protein expression of iRhom2 (**B**) and ADAM17 (**C**) as well as ADAM17 maturation (**D**) were quantified by densitometric analysis. ADAM17 surface expression was determined by flow cytometry (**E**,**F**). An exemplary histogram (**E**) and the quantified specific median fluorescence (**F**) are shown. All quantitative data are displayed as mean + SD of three independent experiments. Statistical differences in comparison to the control (ctrl) are indicated by black asterisks (* = p ≤ 0.05; ** = p ≤ 0.01; *** = p ≤ 0.001).

Since the 6 h of cytokine stimulation was optimal for mRNA induction we expected that effects on the protein level should become visible sometime after 6 h. In fact, after 24 h stimulation with TNFα or co-stimulation with IFNγ and TNFα a significant upregulation of iRhom2 protein expression was detectable by western blotting using a monoclonal antibody against iRhom2 (Fig. 3A,B, suppl. Figure 3A) Stimulation with IFNγ or TNFα alone did not have a significant effect on the protein expression level. Since there exists no reliable antibody against iRhom1 we could not investigate iRhom1 protein expression.

The main function of iRhoms is to associate with ADAM17 and to promote its maturation and surface expression^25,49,50,51^. Therefore, we performed western blot analysis of cell lysates from stimulated and unstimulated cells to study whether the upregulation of iRhom2 associates with the increased presence of mature ADAM17 compared to the proform of ADAM17 (Fig. 3A–D). In fact, co-stimulation increased the maturation level of ADAM17, but there was no significant upregulation of total ADAM17 protein expression at this time point.

Since only mature ADAM17 is transported to the cell surface^23^ we asked whether the increased maturation would result in increased surface expression of ADAM17. Flow cytometric analysis revealed that co-stimulation with IFNγ and TNFα significantly increased ADAM17 surface expression (Fig. 3E,F). By contrast, stimulation with either IFNγ or TNFα alone had a less pronounced effect or no effect, respectively.

To address the question whether the observed regulatory mechanism would also occur in other epithelial cell lines we performed corresponding experiments with the human intestinal epithelial subclone TC7 of Caco-2 cells and the human lung epithelial cell line A549. As shown before for HT-29 cells, we could observe an induction of iRhom2 but not iRhom1 in response to co-stimulation with the cytokines IFNγ and TNFα which correlated with increased maturation of ADAM17 in both Caco-2 (suppl. Figure 4) and A549 (suppl. Figure 5) cells. The upregulation of ADAM17 surface expression was also evidenced for A549 cells (suppl. Figure 5F). Thus, the observed regulatory mechanism for ADAM17 seems to occur in different types of epithelial cell lines even from different organs.

### Upregulation of iRhom2 involves signaling by NF-κB, AP-1 and JAK

**Figure 4:**
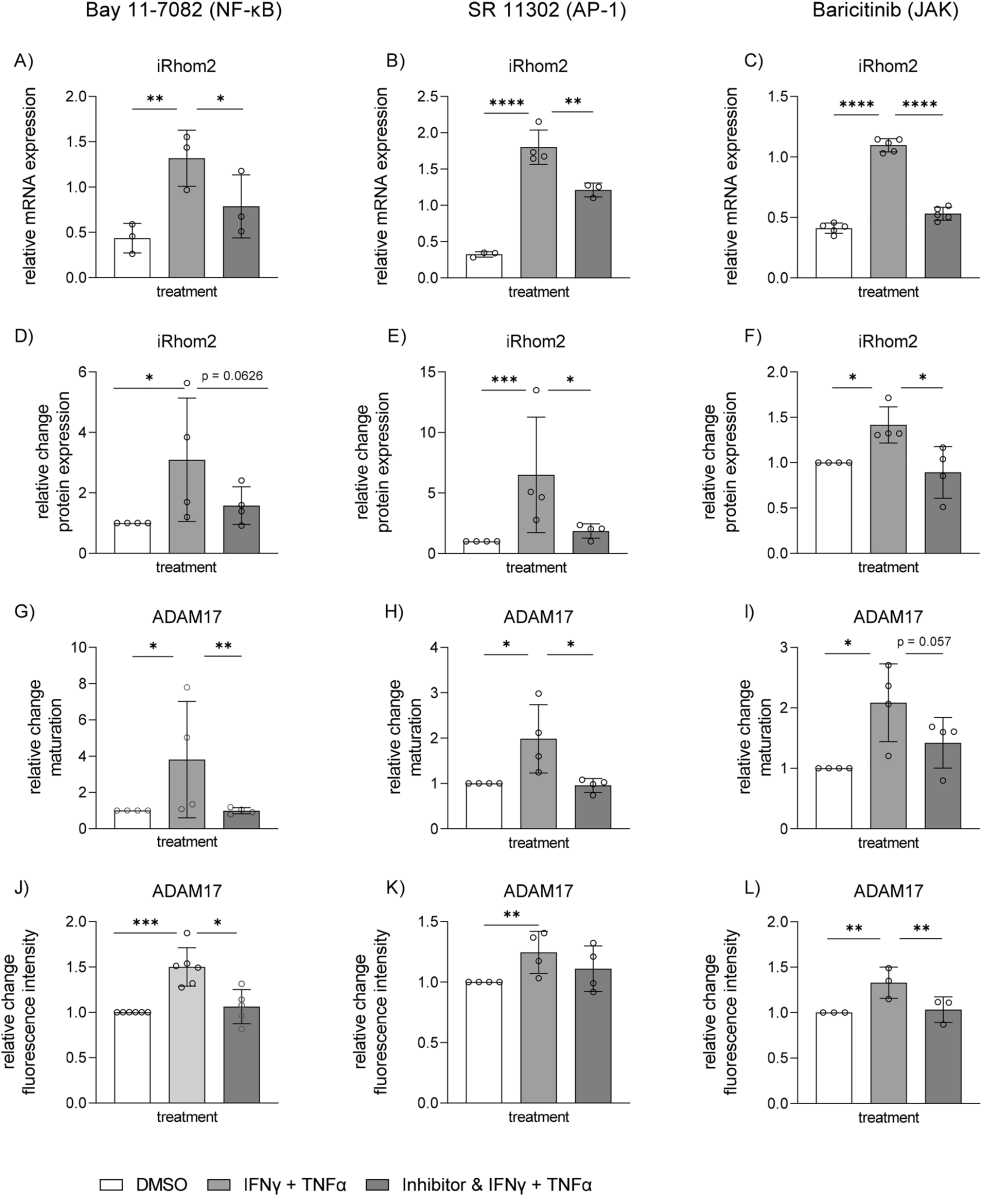
Inhibition of iRhom2 upregulation and ADAM17 regulation. HT-29 cells were treated with the NF-kB inhibitor Bay11-7082 (3 µM), the AP-1 inhibitor SR 11,302 (3 µM), the JAK inhibitor Baricitinib (1 µM) or with the appropriate volume of vehicle control (DMSO). After 1 h, cells were co-stimulated with IFNγ and TNFα (each cytokine 10 ng/ml) or left unstimulated. For analyzing mRNA of iRhom2 by qPCR with GAPDH as reference gene (**A**–**C**), cells were stimulated for 6 h. For the examination of protein levels of iRhom2 with GAPDH as loading control (**D**–**F**) and maturation of ADAM17 by western blot (**G**–**I**) and ADAM17 surface expression by flow cytometry (**J**–**L**) cells were stimulated for 24 h. Quantitative data are displayed as mean + SD of at least three independent experiments. Statistical differences in comparison to IFNγ and TNFα stimulated cells are indicated by asterisks (* = p ≤ 0.05; ** = p ≤ 0.01; *** = p ≤ 0.001).

Since co-stimulation with IFNγ and TNFα could efficiently upregulate iRhom2 expression we next analyzed the involvement of key signaling events that are known to be activated by these cytokines (Fig. 4). We first performed a bioinformatic binding site analysis using CiiiDER^44^ for the putative promoter region of iRhom2 and recognized potential binding sites for NF-κB, AP-1 and STAT1 transcription factors. In fact, there were 4 typical sites for NF-κB proteins, 6 for AP-1 proteins and 1 for the transcription factor STAT1 in iRhom2 (suppl. Figure 3B).

To study whether NF-κB or AP-1 are relevant for transcriptional induction of iRhom2 expression we used the pharmacological inhibitors Bay11-7082 and SR 11302, respectively. Both transcriptional pathways can be triggered by TNFα^52,53^, while only the NF-κB pathway but not the AP-1 pathway is activated by IFNγ^53,54^. To selectively inhibit IFNγ induced signaling we chose Baricitinib which blocks JAK activity that is directly triggered by IFNγ receptors upon ligand binding.

We found that all inhibitors reduced the rise in iRhom2 mRNA (Fig. 4A-C) and protein expression (Fig. 4D-F and suppl. Figure 6D-F) induced by the combined stimulation with IFNγ and TNFα. Noteworthy, the most complete inhibition was observed with Baricitinib, while Bay11-7082 and SR 11302 lead to partial inhibition. Moreover, this inhibition was associated with decreased ADAM17 maturation (Fig. 4G-I) and reduced ADAM17 surface expression (Fig. 4J-L and suppl. Figure 7.) in cells stimulated with both cytokines.

### Effects of iRhom2 upregulation can be mimicked by iRhom2 overexpression

**Figure 5:**
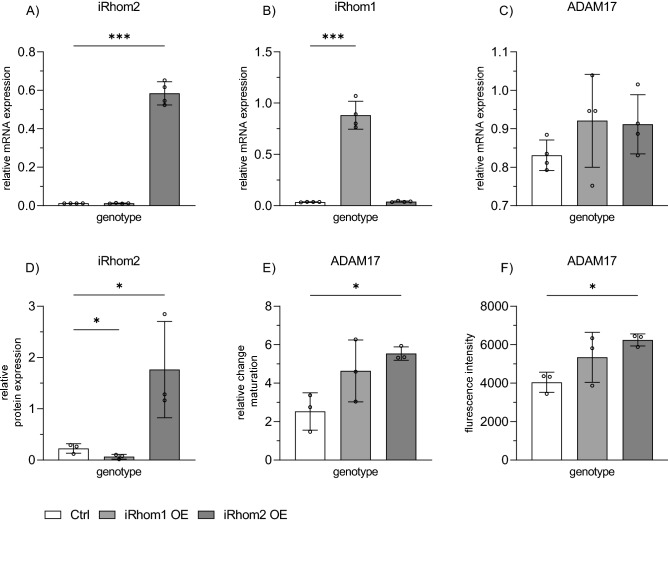
Effect of iRhom2 overexpression on ADAM17 regulation. HT-29 cells were transduced with lentivirus coding for iRhom1 (iRhom1 OE) or 2 (iRhom2 OE) or with control vector (Ctrl). After selection of transduced cells, mRNA expression of iRhom2 (**A**), iRhom1 (**B**), ADAM17 (**C**) was examined by qPCR with GAPDH as reference gene. Protein expression of iRhom2 with GAPDH as loading control and maturation of ADAM17 were analyzed by western blot (**D**,**E**). ADAM17 surface expression was determined by flow cytometry (**F**). Quantitative data are displayed as mean + SD of at least three independent experiments. Statistical differences in comparison to the control (ctrl) are indicated by asterisks (* = p ≤ 0.05; ** = p ≤ 0.01; *** = p ≤ 0.001).

Next, we asked whether overexpression of iRhom2 or iRhom1 would be sufficient to upregulate ADAM17 maturation and surface expression (Fig. 5.). To this end, the cells were transduced with lentivirus encoding human iRhom1 or iRhom2 and overexpression was controlled on the mRNA level for both iRhoms (Fig. 5A, B) and at the protein level for iRhom2 (Fig. 5D and suppl. Figure 8.A). It was confirmed that either iRhom1 or iRhom2 could be overexpressed and specifically detected. Importantly, mRNA expression of neither ADAM17 (Fig. 5C) nor its close relative ADAM10 (suppl. Figure 8B) change with long-term overexpression of iRhom1 or iRhom2. Western blot analysis for ADAM17 protein expression then revealed that overexpression of iRhom2 enhanced maturation of ADAM17 (Fig. 5E and suppl. Figure 8A). Furthermore, flow cytometric analysis confirmed that surface expression of ADAM17 was enhanced as a consequence of this maturation (Fig. 5F and suppl. Figure 8C). By contrast, overexpression of iRhom1 did not significantly affect ADAM17 maturation nor surface expression (Fig. 5E,F). These results indicate that the effects of inflammatory iRhom2 upregulation on ADAM17 maturation and trafficking can be efficiently mimicked by overexpression of iRhom2 but not by overexpression of iRhom1.

### iRhom2 upregulation or overexpression promote ADAM17 dependent shedding

**Figure 6:**
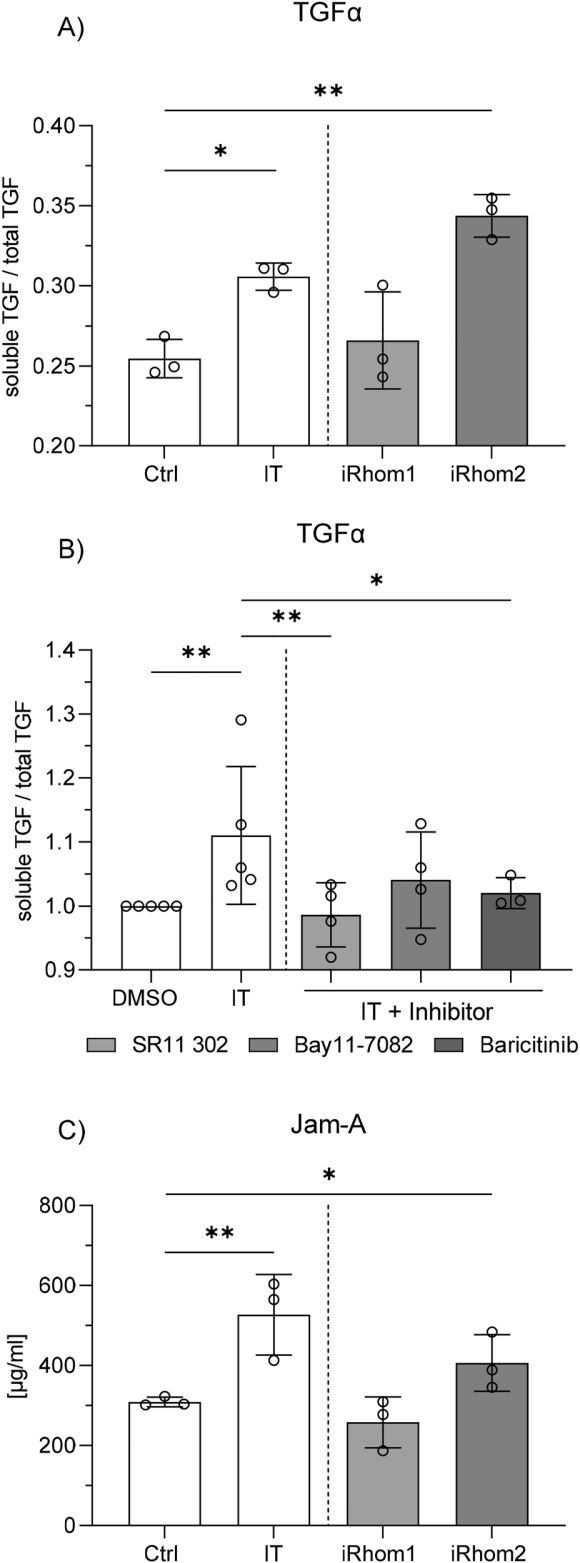
Induction of ADAM17 mediated shedding by iRhom2 upregulation. (**A**): HT-29 cells transduced with lentivirus coding for iRhom1, iRhom2 or control vector were transfected to express a TGFα alkaline phosphatase fusion protein. 24 h after transfection cells were either co-stimulated with IFNγ and TNFα ((IT) each 10 ng/ml) or left untreated. After another 24 h ADAM17 mediated shedding of the fusion protein was determined by a colorimetric assay for phosphatase activity. (**B**): Wildtype HT-29 cells were transfected to express a TGFα alkaline phosphatase fusion protein. 24 h after transfection cells were treated with the NF-kB inhibitor Bay11-7082 (3 µM), the AP1 inhibitor SR11 302 (3 µM), the JAK inhibitor Baricitinib (1 µM) or vehicle control (DMSO) for 1 h before starting the stimulation with IFNγ and TNFα ((IT) each 10 ng/ml). After 24 h, ADAM17 mediated shedding of TGFα-AP was determined by a colorimetric assay. (**C**): HT-29 cells transduced with lentivirus coding for iRhom1, iRhom2 or control vector, either co-stimulated with IFNγ and TNFα ((IT) each 10 ng/ml) for 24 h or left untreated, were analyzed by ELISA for ADAM17 mediated release of cell expressed JAM-A.

To assess how changes in the expression level of iRhoms would affect ADAM17-mediated shedding we performed a shedding assay for TGFα which is a well-established substrate of ADAM17 and is not significantly shed by other related proteases^14^. For easy and reliable quantification of TGFα shedding, cells were transfected with TGFα N-terminally fused to an alkaline phosphatase. This engineered substrate could be enzymatically detected in the cell lysate and in the supernatant. TGFα was constitutively shed in unstimulated and untransfected cells. Induction of iRhom2 expression by co-stimulation with IFNγ and TNFα was associated with increased TGFα shedding, which could be mimicked by overexpression of iRhom2 but not iRhom1 (Fig. 6A). Moreover, this effect was suppressed when the transcriptional induction of iRhom2 after co-stimulation with IFNγ and TNFα was prevented with AP-1- (SR 11302) or JAK- (Baricitinib) inhibitors (Fig. 6B). A reduction of TGFα release was also noted with the NF κB inhibitor (Bay11-7082) but this did not reach significance. These data indicate that increased presence of iRhom2 leads to increased ADAM17 activity. To finally investigate the release of an endogenous substrate we chose the junctional adhesion molecule JAM-A which is constitutively expressed by epithelial cells and is a confirmed substrate of ADAM17^8^. The release of shed, soluble JAM-A into the supernatant of HT-29 cells was determined by ELISA (Fig. 6C). As previously detected for TGFα, the induced iRhom2 expression after co-stimulation with IFNγ and TNFα led to increased shedding of JAM-A. This could be mimicked by the overexpression of iRhom2 but not iRhom1. Therefore, an increased iRhom2 expression level clearly affects physiological ADAM17 substrates with known critical functions in maintaining cell–cell contacts in IBD^18,31,55^.

## Discussion

The present study demonstrates that iRhom2 but not iRhom1 is upregulated in intestinal tissue of mice undergoing prolonged DSS induced colitis. The cytokines IFNγ and TNFα synergistically upregulate iRhom2 mRNA and protein expression in the colon epithelial cell line HT-29. By contrast, iRhom1 is constitutively expressed and not further upregulated by these cytokines. The upregulation of iRhom2 can be reduced by either inhibition of Janus kinases, NF-κB or AP-1. The iRhom2 upregulation is associated with enhanced maturation and surface expression of ADAM17. These effects can be mimicked by overexpression of iRhom2 but not by overexpression of iRhom1. Finally, we demonstrate that enhanced iRhom2 expression by either cytokine stimulation or overexpression results in increased cleavage of the ADAM17 substrates TGFα and JAM-A. Our data therefore indicate that inflammatory iRhom2 upregulation is instrumental to further mobilize intracellularly stored immature ADAM17 and to increase ADAM17 shedding activity.

Previous research has demonstrated that iRhom2 is critical for ADAM17 maturation in immune cells^25,31,49,36,50^. In tissue cells under basal conditions, however, iRhom2 is expressed at a low level and iRhom1 seems to fulfill this function for ADAM17. Consistent with this theory, our bioinformatic analysis demonstrated that iRhom2 is expressed at a low constitutive level in colon epithelial cells whereas iRhom1 is present at a much higher expression level already in unstimulated cells. However, under inflammatory conditions the situation is very different. Here, iRhom2 becomes significantly upregulated and therefore it plays a significant role for the upregulation of ADAM17 maturation, surface expression and activity in colon epithelial cells undergoing inflammatory stimulation. A regulatory mechanism which has also been demonstrated in endothelial cells after inflammatory stimulation^46^. Thus, our findings significantly extend the current knowledge on the specific task of both iRhoms for promoting ADAM17 function by demonstrating that iRhom2 is not only relevant in immune cells but also in epithelial tissue cells under inflammatory conditions.

As explained above, iRhom1 is expressed already in resting cells where iRhom2 is only weakly expressed. Thus, at this stage constitutive ADAM17 maturation should be mainly due to iRhom1. Overexpression of iRhom1 only slightly enhances ADAM17 maturation. But this is not significant and not comparable to the effect of iRhom2 overexpression. This could be due to differences between both iRhoms in their trafficking mechanism towards ADAM17, in their affinity for ADAM17, in their stability after complex formation with ADAM17 or in their susceptibility to an unknown inhibition mechanism. Further research needs to determine whether such differences reside in the N-terminal part of iRhoms showing the lowest degree of homology between both proteins^56^.

IFNγ and TNFα are well known to promote the development of IBD mostly by induction of inflammatory transcriptional pathways. We found that the expression of iRhom2 is considerably upregulated by co-stimulation with these two cytokines. Interestingly, either cytokine alone has no or only weak effects on iRhom2 expression. These data indicate a cooperation of signaling pathways of the two cytokines. Synergism of both cytokines has also been reported in DSS induced colitis^57^. We observed that the cooperative induction of iRhom2 expression by IFNγ and TNFα is almost completely switched off by blocking the JAK activity downstream of the IFNγ receptor with Baricitinib. These results indicate the importance of JAK for inducing iRhom2-dependent ADAM17 activity in an inflammatory setting. They also indicate that this approved inhibitor routinely used for treatment of chronic inflammatory diseases is capable to limit the activity of this protease. Our bioinformatic promoter analysis indicates that both NF-κB and AP-1 can be involved in iRhom2 induction and in fact this was confirmed using inhibitors for either transcription factor. Interestingly NF-κB can be directly switched on by IFNγ^54,58,59^ as well as by TNFα^60,61^. However, it seems likely that the binding of NF-κB to the few sites is not sufficient for iRhom2 induction and that more efficient induction occurs when the AP-1 pathway is also triggered via TNFα, thereby promoting AP-1 binding to the iRhom2 promoter. To study this possibility in more detail and to gain more insight on the individual binding sites as well as other possible pathways, a mutational in-depth promoter analysis is necessary. In addition, posttranscriptional mechanisms may be implicated leading to enhanced presence of iRhom2 protein. It may be possible that TNFα stimulation leads to stabilization of iRhom2 mRNA similarly as it has been described for CX3CL1 mRNA^62^.

IBD is driven by several mediators of chronic inflammation and increasing evidence indicates that the impairment of the epithelial barrier function in the gut is a crucial pathogenic factor. In this setting the iRhom/ADAM17 axis may play an ambivalent role, by activating protective pathways important for regeneration as well as by inducing inflammatory signaling. Protective pathways can be triggered by the shedding of growth factors which then transactivate EGFR and by this mediate epithelial regeneration^5,9,18,63,64,65^. The importance of ADAM17 for this process has been demonstrated using mice with severely reduced ADAM17 expression which suffer from increased barrier disruption. In normal gut tissue, the basal presence of ADAM17 and iRhom1 expression appears to be sufficient to maintain basal shedding activity providing growth signals for tissue regeneration. Under inflammatory conditions however, iRhom2 upregulation would promote additional ADAM17 activity and this would generate an excess of growth. It is not known whether the basal iRhom1 expression is sufficient to cope with the increased need of tissue regeneration. Interestingly, an iRhom2 mutation leading to reduction of function did not affect sensitivity to DSS induced weight loss, implying that iRhom2 was physiologically redundant for the processing of EGFR ligands^50^.

ADAM17 is critical for the release of soluble TNFα which is a crucial driver of the inflammatory disease. In fact, by tissue specific knockout, epithelial ADAM17 has been shown to promote intestinal inflammation via the shedding of TNFα. Moreover, ADAM17 sheds JAM-A on epithelial cells, which is important for epithelial tight junction formation and thus for maintenance of the mucosal barrier^17^. Increased shedding of JAM-A driven by the iRhom2/ADAM17 axis may accelerate the inflammatory process. Of note, we observed a more pronounced effect of increased iRhom2 expression on JAM-A shedding than TGFα shedding. This may be due to the different expression level and subcellular localization of the two substrates resulting in different susceptibility towards ADAM17 and different shedding kinetics. Although this may also indicate more potential long term relevance of JAM-A shedding it remains to be determined which shedding pathway is in fact contributing to disease development in vivo. Several more shedding events such as shedding of cadherins could also be implicated in this scenario. In fact, IFNγ drives IBD pathogenesis through VE-cadherin–directed vascular barrier disruption^66^ and this could potentially be mediated by proteolytic shedding.

Because of its ambivalent role, general inhibition of ADAM17 does not appear to be reasonable in the treatment of chronic inflammatory diseases such as rheumatoid arthritis or IBD. As one potential strategy to limit the inflammatory activities more specifically and to spare physiological activities it has been suggested to develop iRhom2-specific inhibitors of ADAM17. These inhibitors would still allow iRhom1-dependent ADAM17 activity in non-inflamed tissue, for instance, growth factor driven regeneration. The findings of our present study suggest that induction of iRhom2 in inflamed gut epithelial cells significantly contributes to the upregulated ADAM17 dependent mediator release. This upregulation pathway could be selectively prevented by iRhom2 specific inhibitors allowing more selective targeting of inflammatory activities of ADAM17.

## Supplementary Information

Supplementary Figures.

## Data Availability

The original datasets generated or analyzed during the present study are available from the corresponding author on reasonable request.
