# Supplementary material for: Inflammatory activation of surface molecule shedding by upregulation of the pseudoprotease iRhom2 in colon epithelial cells
Source: Sci Rep. 2021 Dec 20;11:24230. doi: 10.1038/s41598-021-03522-2 (PMC8688420; doi:10.1038/s41598-021-03522-2)
Supplement: Supplementary file 1 — Supplementary Figures. [file 41598_2021_3522_MOESM1_ESM.pdf]

Inflammatory activation of surface molecule shedding by upregulation of the pseudoprotease iRhom2 in colon epithelial cells

Anja Adelina Giese*[1], Aaron Babendreyer*[1], Peter Krappen[1], Annika Gross[2], Pavel Strnad[2], Stefan Düsterhöft[#1], Andreas Ludwig[#1]

[1] Institute of Molecular Pharmacology, Medical Faculty, RWTH Aachen University, Aachen, Germany

[2] Division of Gastroenterology and Hepatology, Department of Medicine III, University Hospital RWTH Aachen, Aachen, Germany

[#] *contributed equally

Running title: Inflammatory iRhom2 induction

Address correspondence to:

Andreas Ludwig,

Institute of Molecular Pharmacology,

RWTH Aachen University, Pauwelsstr. 30, 52074 Aachen, Germany;

Phone: +49 241 8035771, Fax: +49 241 8082433, E-Mail: aludwig@ukaachen.de

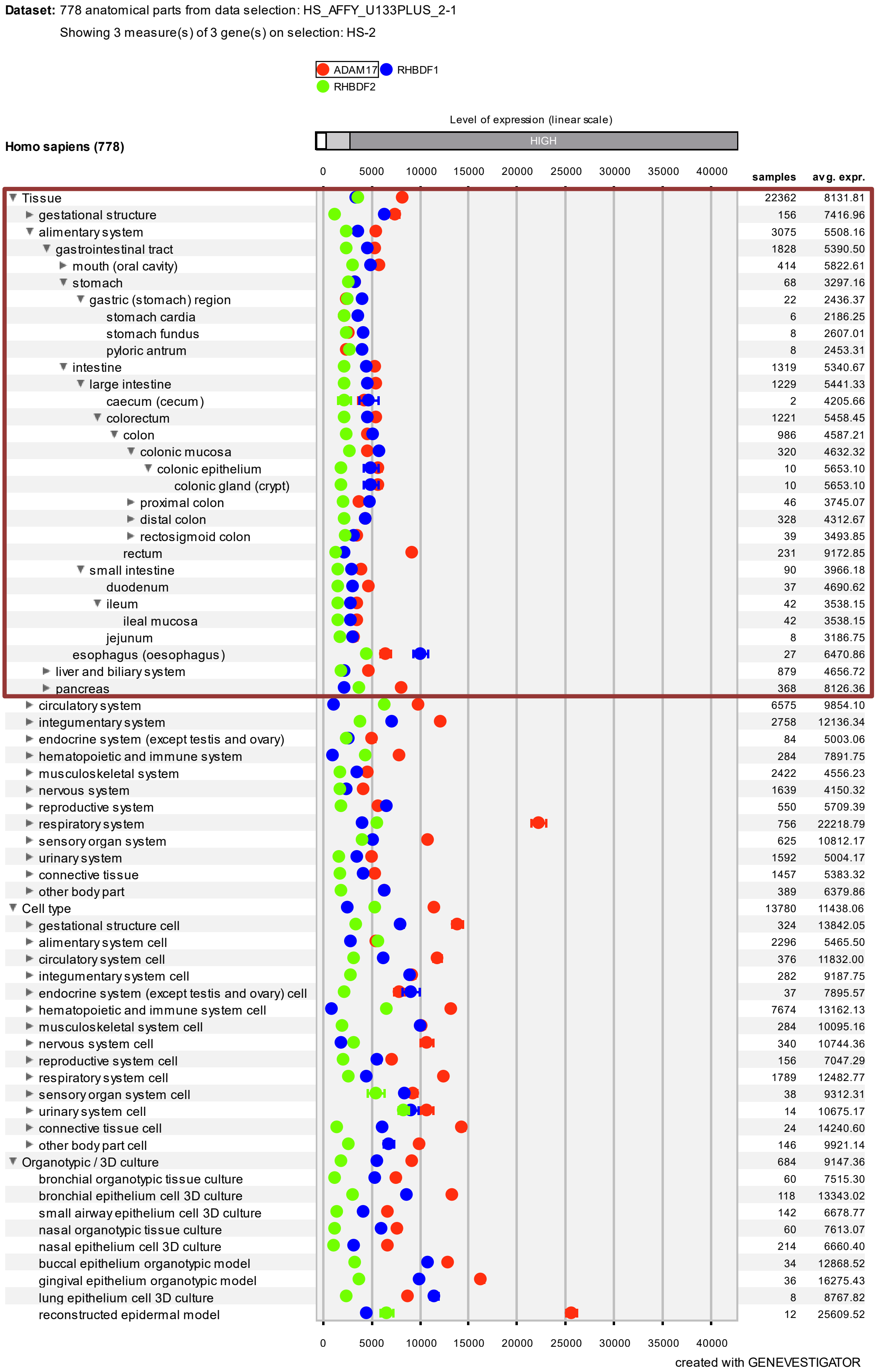

Supplementary Figure 1: Bioinformatic analysis of public transcriptomic data.

Using the GENEVESTIGATOR® suite transcriptome data from human samples generated with Affymetrix Human Genome U133Plus 2.0 were analyzed. Expression patterns of ADAM17 (red), iRhom2 (green) and iRhom1 (blue) mRNA in the data sets of untreated samples are presented. Samples from gastrointestinal samples are boxed in red.

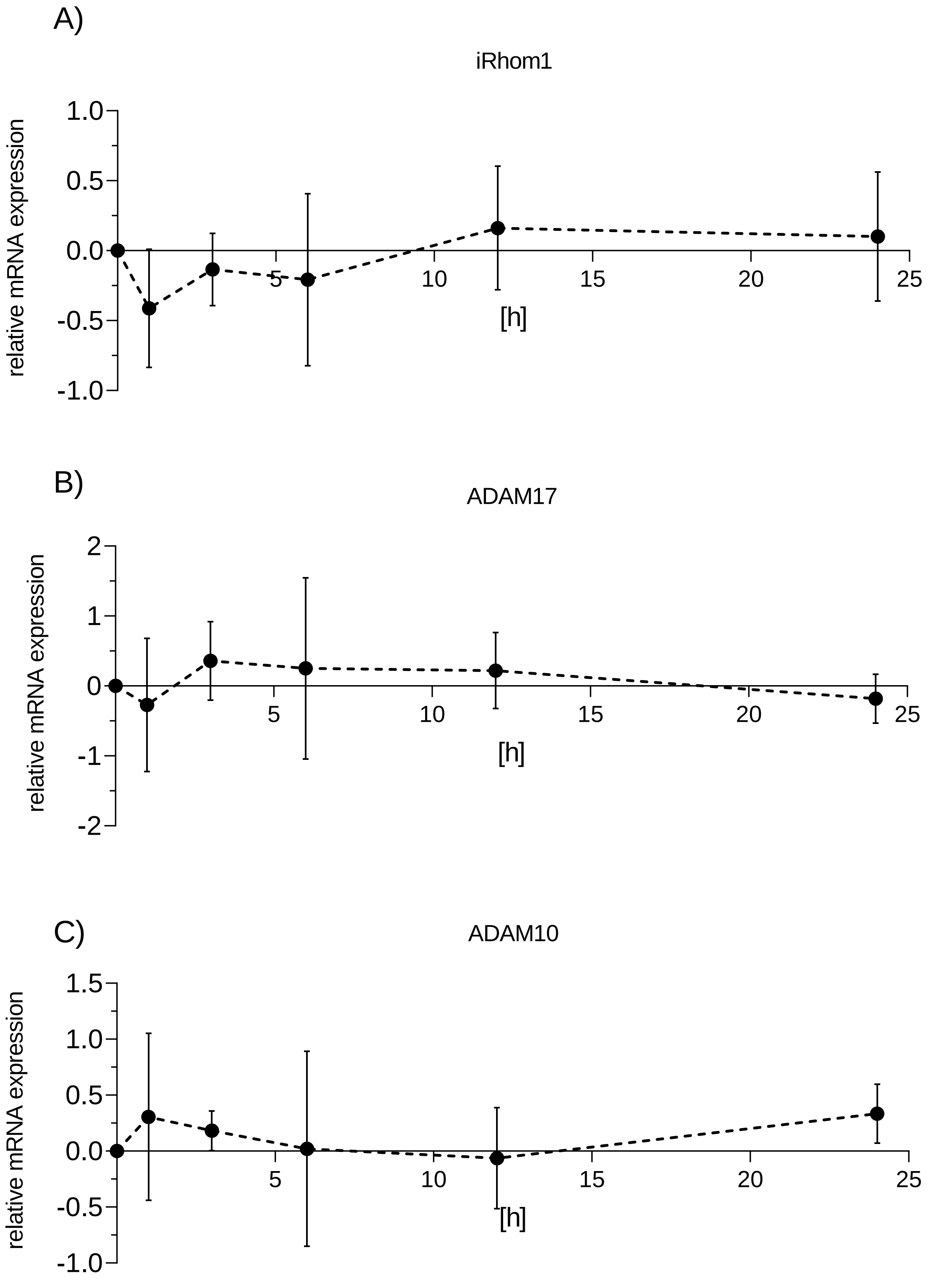

Supplementary Figure 2.: Co-stimulation with IFNγ and TNFα does not not induce mRNA expression of iRhom1, ADAM17 or ADAM10

mRNA expression of iRhom1 (A), ADAM17 (B) and ADAM10 (C) was analyzed by qPCR with GAPDH as reference gene. The values shown indicate the expression of iRhom1, ADAM17 and ADAM10 minus the basal expression at each indicated time point. Data are shown as mean +SD of at least three independent experiments. Statistical differences in comparison to the control (Ctrl) are indicated by asterisks (* = $p \leq 0.05$; ** = $p \leq 0.01$; *** = $p \leq 0.001$).

A)

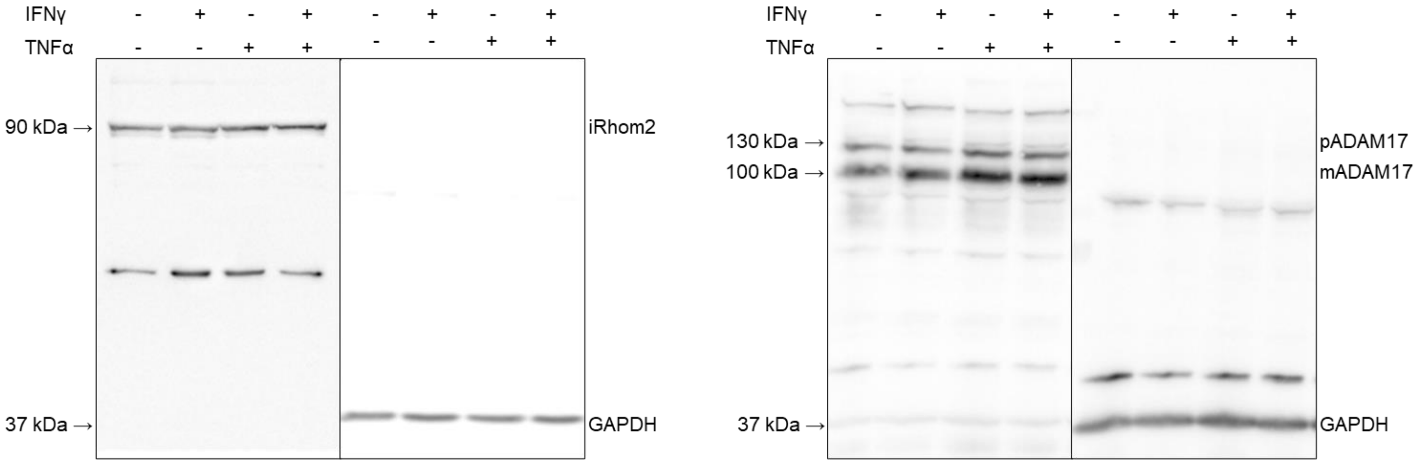

B)

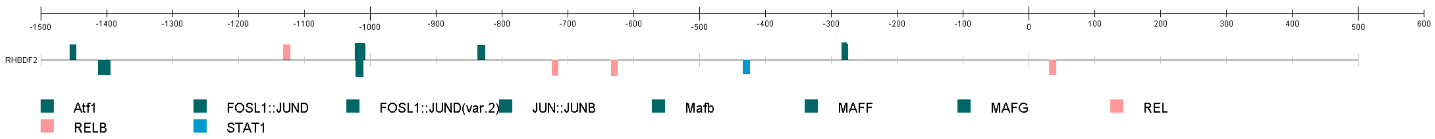

Supplementary Figure 3:

(A): Uncropped images for western blot detection of ADAM17, iRhom2 and GAPDH. Results correspond to the experiment shown in Figure 3. All results are derived from the same gel. A representative experiment is shown which was reproduced three times.

(B): Predicted binding sites of AP-1, NF-κB and STAT-1 transcription factors within in the promoter region of iRhom2 Using the UCSC Genome Browser the promoter region of the RHBDF2 gene was defined as -1500 bp TSS +500 bp. Afterwards potential binding sites of AP-1 (green), NF-κB (rosé) and STAT-1 (blue) transcription factors were defined with Ciiider.

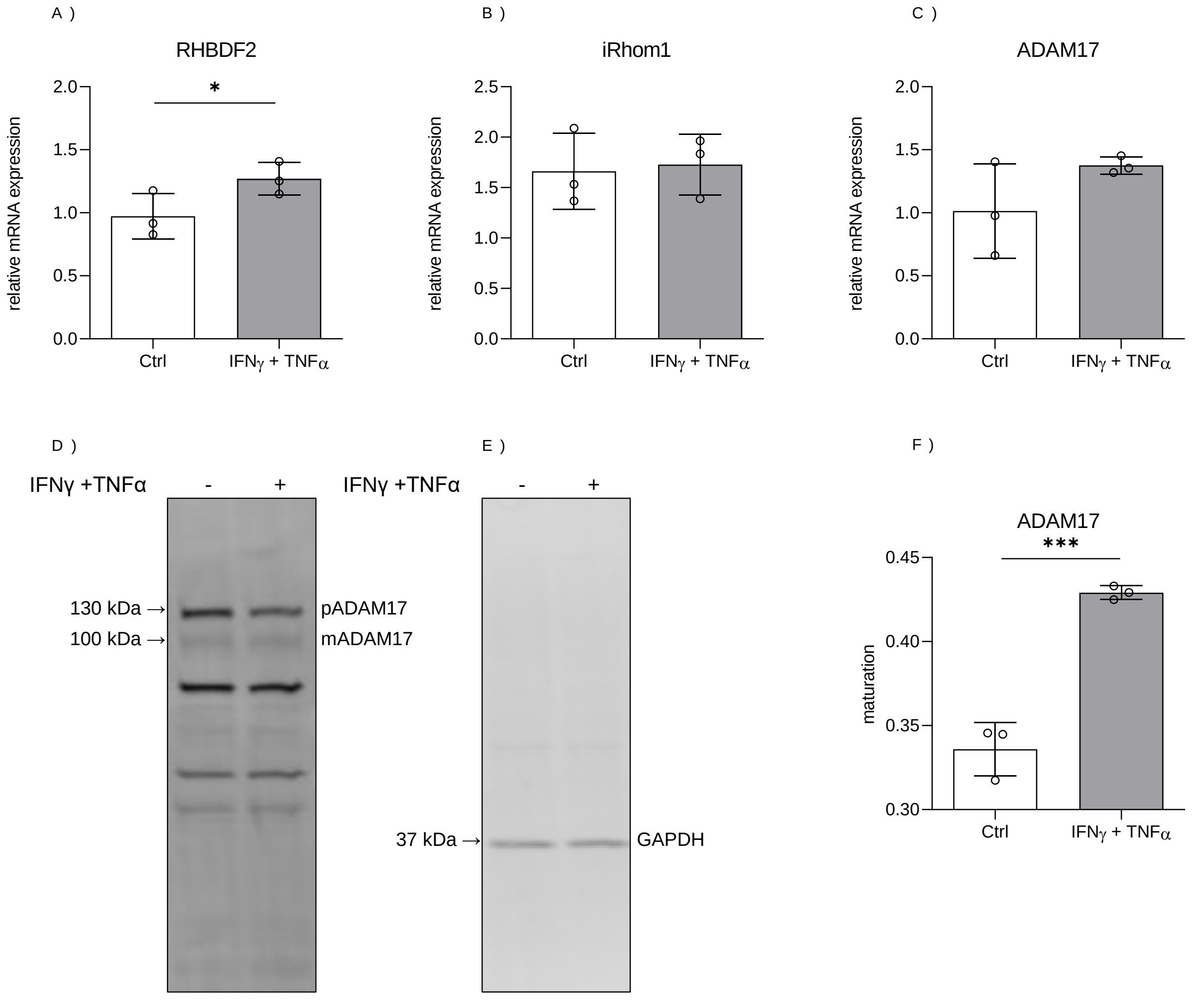

Supplementary Figure 4: Co-stimulation with IFNγ and TNFα leads to increased iRhom2 protein expression and ADAM17 maturation in colorectal epithelial Caco2 cells

Caco2 cells were left unstimulated (Ctrl) or were co-stimulated with IFNγ and TNFα (each cytokine 10 ng/ml) (6 h for gene expression analysis; 24 h for protein level analysis). After mRNA isolation, expression of iRhom2 (A), iRhom1 (B) and ADAM17 (C) was analyzed with GAPDH as reference gene. Maturation of ADAM17 was analyzed by western blot with GAPDH as loading control and quantified by densitometric analysis. (F). An exemplary western blot of ADAM17 and GAPDH from the same gel is shown (D, E). All quantitative data are displayed as mean +SD of three independent experiments. Statistical differences in comparison to the control (Ctrl) are indicated by black asterisks (* = p ≤ 0.05; ** = p ≤ 0.01; *** = p ≤ 0.001).

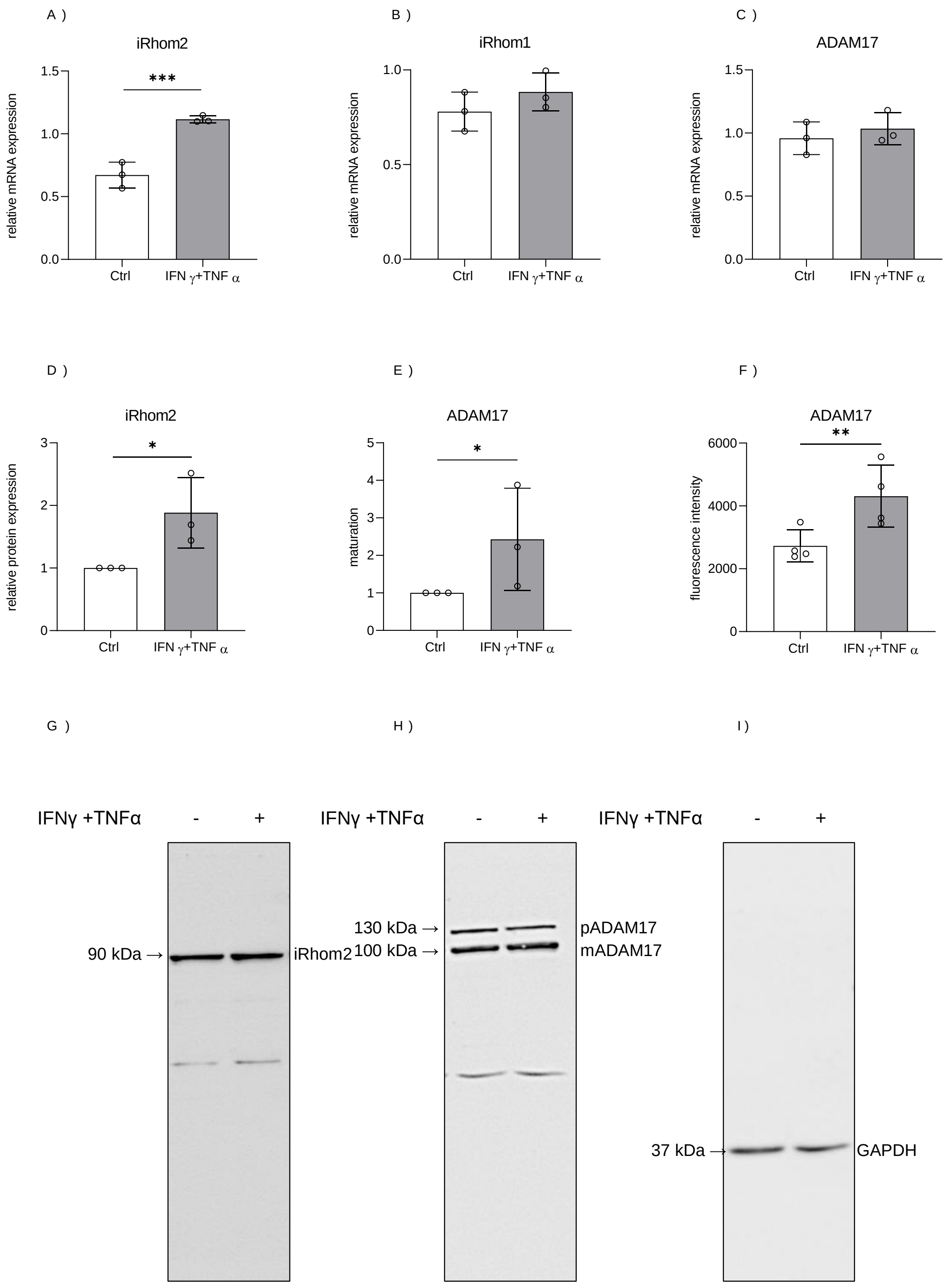

Supplementary Figure 5: Co-stimulation with IFNγ and TNFα leads to increased iRhom2 protein expression, ADAM17 maturation and surface expression in lung epithelial A549 cells

A549 cells were left unstimulated (Ctrl) or co-stimulated with IFNγ and TNFα (each cytokine 10 ng/ml) (6 h for gene expression analysis; 24 h for protein level analysis). After mRNA isolation, expression of iRhom2 (A), iRhom1 (B) and ADAM17 (C) was analyzed with GAPDH as reference gene. Protein level of iRhom2 and maturation of ADAM17 were analyzed by western blot with GAPDH as loading control and quantified by densitometric analysis. (D, E). Surface expression of ADAM17 was determined by flow cytometry and quantified as specific median fluorescence intensity (F). An exemplary western blot of iRhom2, ADAM17 and GAPDH from the same gel is shown (G, H, I). All quantitative data are displayed as mean +SD of three independent experiments. Statistical differences in comparison to the control (Ctrl) are indicated by black asterisks (* = $p \leq 0.05$; ** = $p \leq 0.01$; *** = $p \leq 0.001$).

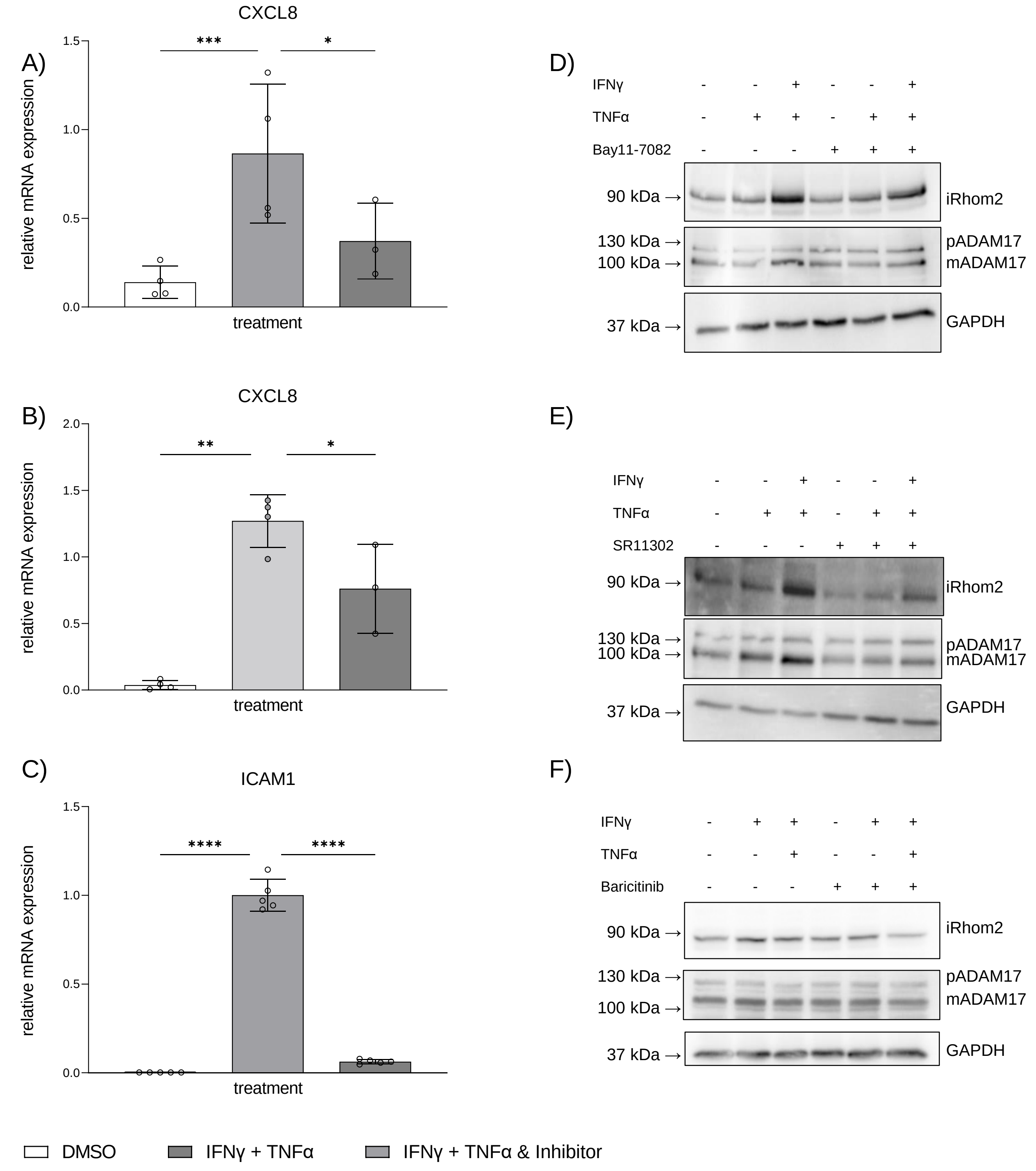

Supplementary Figure 6: NF-κB- (Bay11-7082), AP-1- (SR11302) and JAK- (Baricitinib) inhibitors reduce iRhom2 protein expression and ADAM17 maturation

HT-29 cells were left untreated or treated with the NF-kB inhibitor Bay11-7082 (3 µM), the AP-1 inhibitor SR 11302 (3 µM), the JAK inhibitor Baricitinib (1 µM) or vehicle control (DMSO). After 1 h, cells were co-stimulated with IFNγ and TNFα (each cytokine 10 ng/ml) or left unstimulated. To assess the efficacy of the inhibitors, the mRNA expression of typical target genes (IL-8 for NF- κB and AP-1 and ICAM for STAT-1) were analyzed with qPCR and GAPDH as reference gene after 6 h of stimulation (A-C) . The examination of protein levels of iRhom2, ADAM17 and maturation of ADAM17 were performed after 24 h of stimulation. Exemplary western blots of three independent experiments are shown in D-F. **Uncropped western blots for iRhom2, ADAM17 and GAPDH can be found in Supplementary Figure 9**. Quantitative data are displayed as mean +SD of at least three independent experiments. Statistical differences in comparison to the control (ctrl) are indicated by asterisks (* = p ≤ 0.05; ** = p ≤ 0.01; *** = p ≤ 0.001).

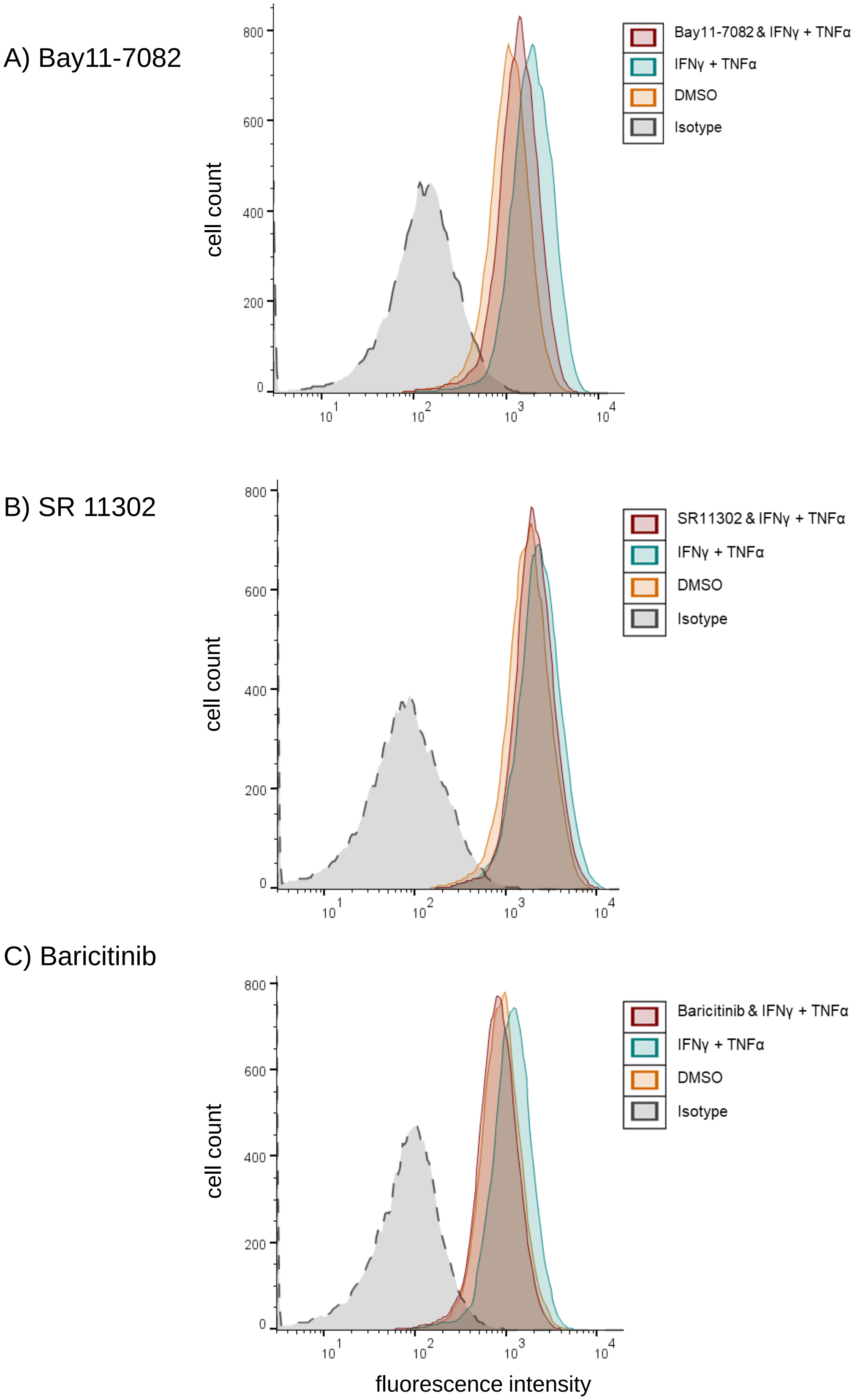

Supplementary Figure 7: Effect of NF-κB- (Bay11-7082), AP-1- (SR 11302) and JAK- (Baricitinib) on upregulation of ADAM17 surface expression in response to IFNγ and TNFα.

HT-29 cells were left untreated or treated with the NF-kB inhibitor Bay11-7082 (3 µM), the AP-1 inhibitor SR 11302 (3 µM), the JAK inhibitor Baricitinib (1 µM) or vehicle control (DMSO). After 1 h, cells were co-stimulated with IFNγ and TNFα (each cytokine 10 ng/ml) or left unstimulated. Surface expression of ADAM17 was studied by flow cytometry. Unspecific fluorescence was determined by an isotype control antibody. Exemplary histograms of three independent experiments are shown.

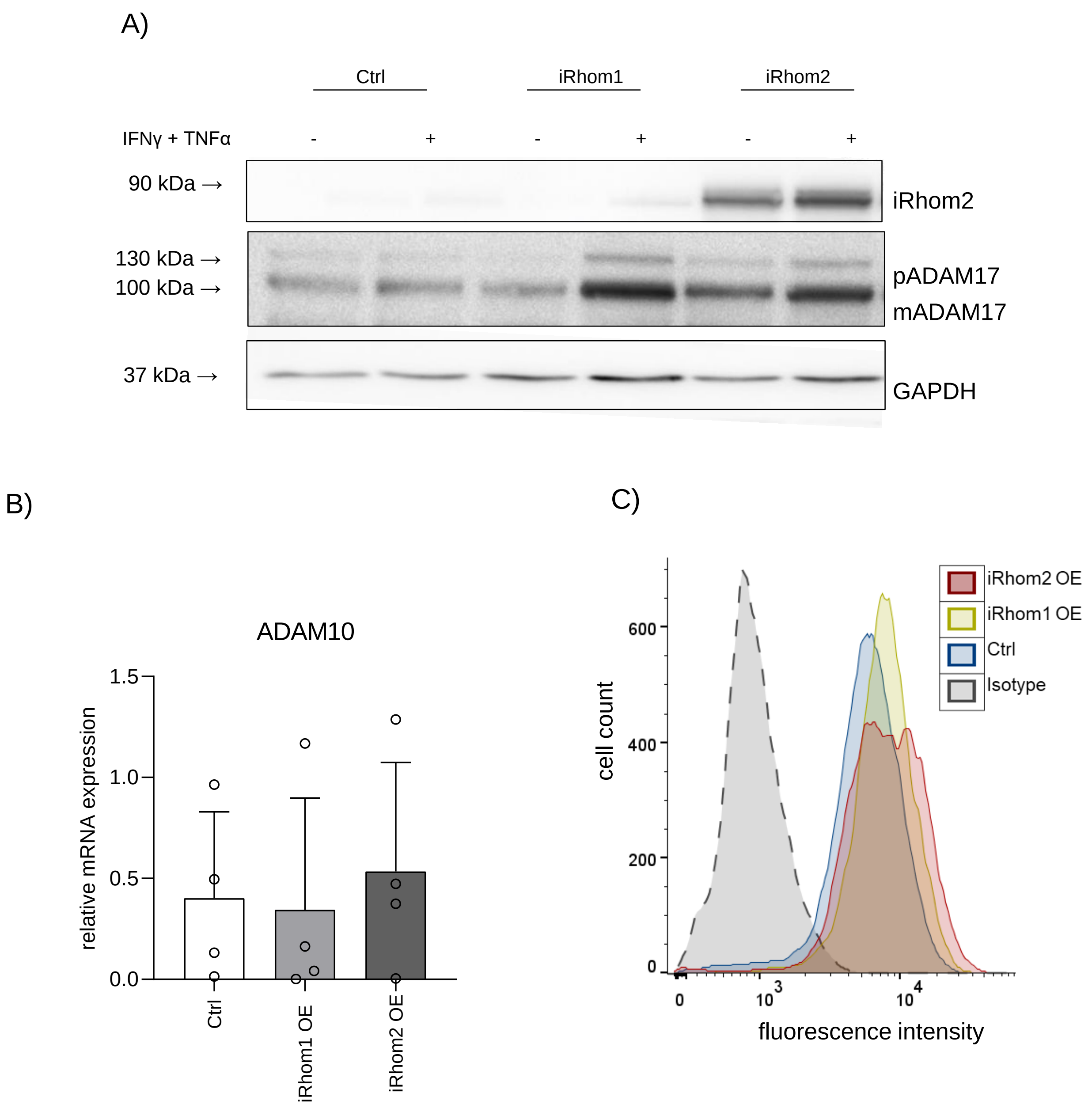

Supplementary Figure 8 : Long-term overexpression of iRhom2 leads to a higher maturation rate of ADAM17 in HT-29 cells

HT-29 cells were transduced with lentivirus coding for iRhom1 or 2 or with control vector. After selection cells were co-stimulated with IFNγ and TNFα (each cytokine 10 ng/ml) for 24 h or left unstimulated. **A:** ADAM17 maturation and protein levels of iRhom2 were determined by western blotting with GAPDH as loading control. An exemplary western blot is shown**. Uncropped western blots of iRhom2, ADAM17 and GAPDH can be found in Supplementary Figure 10**. **B:** mRNA expression of ADAM10 was examined by qPCR with GAPDH as reference gene. **C:** ADAM17 was studied by flow cytometry. Unspecific fluorescence was determined by an isotype control antibody. An exemplary histogram of there independent experiments is shown. Quantitative data are displayed as mean +SD of at least three independent experiments. Statistical differences in comparison to the control (ctrl) are indicated by asterisks (* = $p \le 0.05$; ** = $p \le 0.01$; *** = $p \le 0.001$).

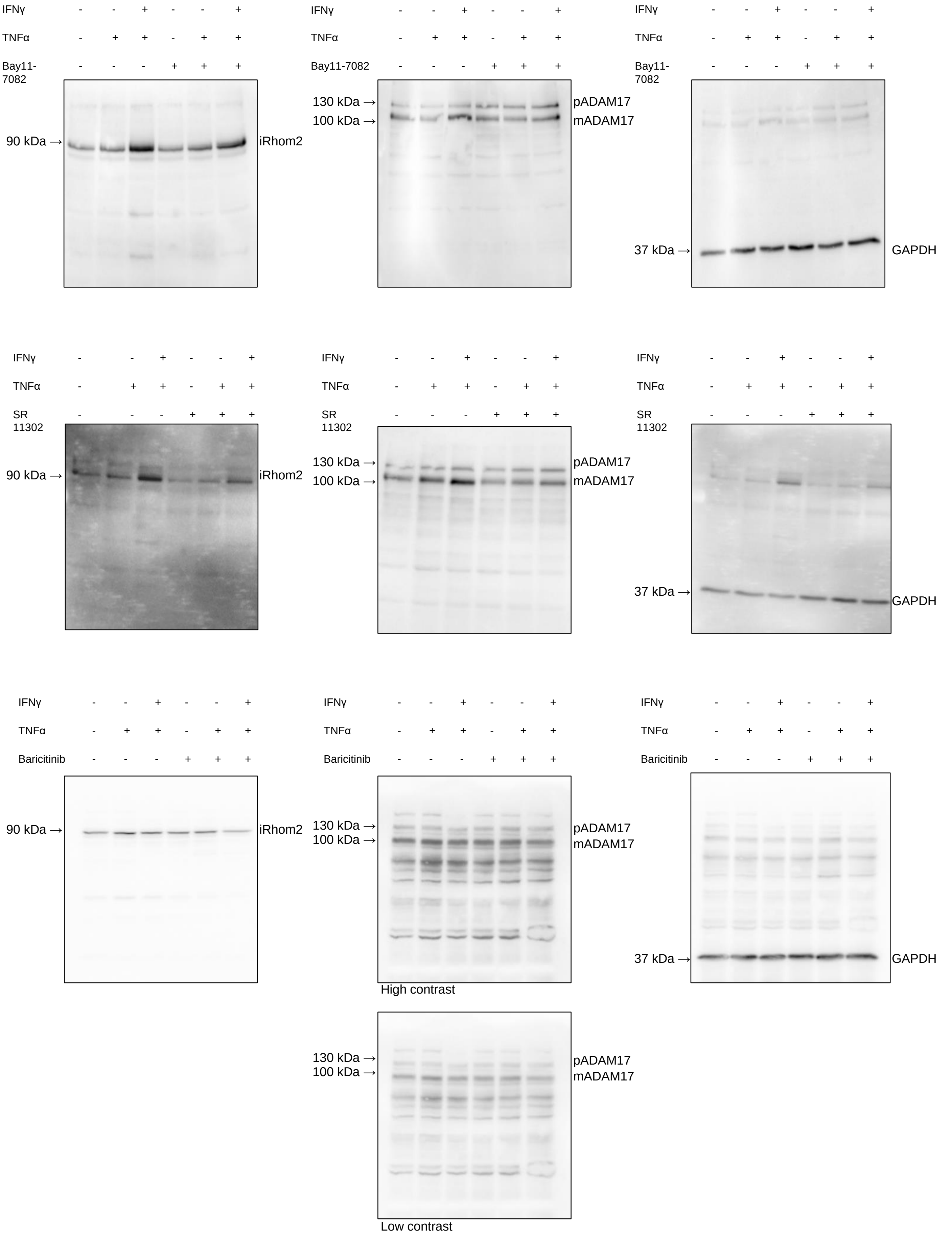

Supplementary Figure 9: Uncropped images for western blot detection of ADAM17, iRhom2 and GAPDH of Supplementary Figure 6 *NF-κB- (Bay11-7082), AP-1- (SR11302) and JAK- (Baricitinib) inhibitors reduce iRhom2 protein expression and ADAM17 maturation*

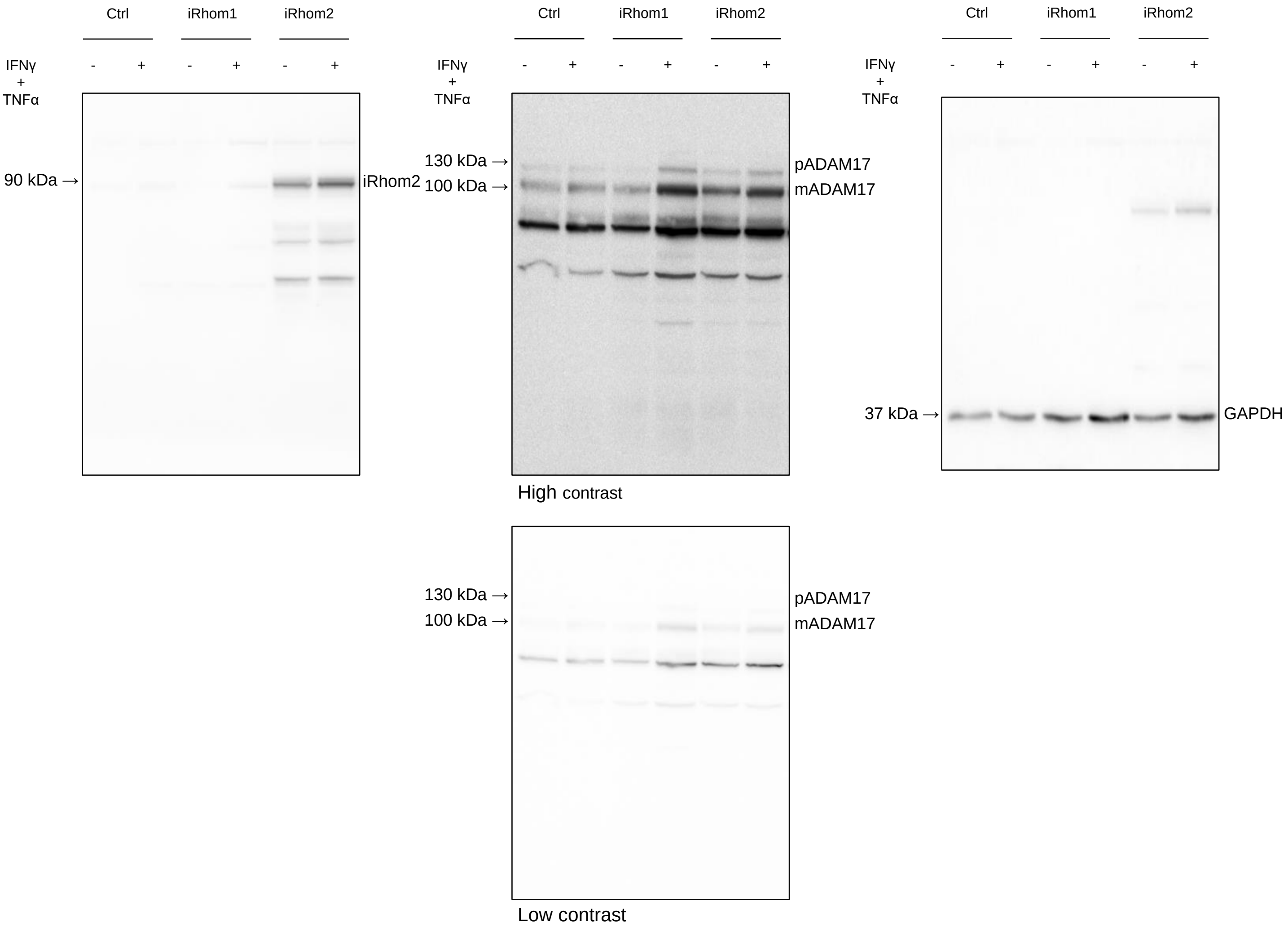

Supplementary Figure 10: Uncropped images for western blot detection of ADAM17, iRhom2 and GAPDH of Supplementary Figure 8: *Long-term overexpression of iRhom2 leads to a higher maturation rate of ADAM17 in HT-29 cells*
